# Swedish mutant APP-based BACE1 binding site peptide reduces APP β-cleavage and cerebral Aβ levels in Alzheimer’s mice

**DOI:** 10.1038/srep11322

**Published:** 2015-06-19

**Authors:** Song Li, Huayan Hou, Takashi Mori, Darrell Sawmiller, Adam Smith, Jun Tian, Yanjiang Wang, Brian Giunta, Paul R. Sanberg, Sheqing Zhang, Jun Tan

**Affiliations:** 1 Department of Psychiatry and Behavioral Neurosciences, Morsani College of Medicine, University of South Florida, Tampa, FL 33613; 2 Center for Translational Research of Neurology Diseases, First Affiliated Hospital, Dalian Medical University, Dalian 116011, China; 3 Departments of Biomedical Sciences and Pathology, Saitama Medical Center and Saitama Medical University, Kawagoe, Saitama 350-8550, Japan; 4 Department of Neurosurgery and Brain Repair, Center Of Excellence for Aging & Brain Repair, Morsani College of Medicine, University of South Florida, Tampa, FL 33613; 5 Department of Neurology, Daping Hospital, the Third Military Medical University, Chongqing 400042, China; 6 Neuroimmunology Laboratory, Department of Psychiatry and Behavioral Neurosciences, Morsani College of Medicine, University of South Florida, Tampa, FL 33613; 7 Department of Neurology, Changhai hospital, Shanghai 200433, China

## Abstract

BACE1 initiates amyloid-β (Aβ) generation and the resultant cerebral amyloidosis, as a characteristic of Alzheimer’s disease (AD). Thus, inhibition of BACE1 has been the focus of a large body of research. The most recent clinical trials highlight the difficulty involved in this type of anti-AD therapy as evidenced by side effects likely due to the ubiquitous nature of BACE1, which cleaves multiple substrates. The human Swedish mutant form of amyloid protein precursor (APPswe) has been shown to possess a higher affinity for BACE1 compared to wild-type APP (APPwt). We pursued a new approach wherein harnessing this greater affinity to modulate BACE1 APP processing activity. We found that one peptide derived from APPswe, containing the β-cleavage site, strongly inhibits BACE1 activity and thereby reduces Aβ production. This peptide, termed APPswe BACE1 binding site peptide (APPsweBBP), was further conjugated to the fusion domain of the HIV-1 Tat protein (TAT) at the C-terminus to facilitate its biomembrane-penetrating activity. APPwt and APPswe over-expressing CHO cells treated with this TAT-conjugated peptide resulted in a marked reduction of Aβ and a significant increase of soluble APPα. Intraperitoneal administration of this peptide to 5XFAD mice markedly reduced β-amyloid deposits as well as improved hippocampal-dependent learning and memory.

The proteolysis of the type 1 membrane-anchored amyloid precursor protein (APP) by the sequential actions of β- and γ-secretases results in amyloid-β (Aβ) peptide production that is thought to be causal for Alzheimer’s disease (AD)^1^,^2^,^3^,^4^. Inhibition or modulation of β- and/or γ-secretases constitutes important therapeutic strategies for AD and have become the centerpiece of therapeutically oriented research on this disease.

Presenilin 1 and 2 (PS1/PS2), two integral membrane proteins found in the endoplasmic reticulum and Golgi apparatus, are the major enzymatic targets for γ-secretase inhibition for the treatment of AD^5^. However, apart from their roles in AD, PS1/PS2 also controls the Notch signaling pathway responsible for cell proliferation and differentiation during embryonic development^6^. PS1/PS2-null mice have massive neuronal loss, skeletal defects, underdeveloped subventricular areas and severe hemorrhages, and only a few types of PS1/PS2-null mouse models survive after birth^7^,^8^,^9^,^10^. Other substrates of PS1/PS2 have also been identified, suggesting pleotropic function of the PSs^11^. Most importantly, recent clinical trials have indicated that inhibition of γ-secretase is likely to cause undesirable side effects^12^. Indeed, several such inhibitors, including avagacestat (Bristol-Myers Squibb), tarenflurbil (Flurizan, Myriad Genetics) and semagacestat (Eli Lilly and Co.), have failed to complete Phase III clinical trials^12^,^13^,^14^,^15^,^16^. In the case of semagacestat, activities of daily living and cognition even worsened in the treated patients^14^,^15^,^16^.

Like γ-secretase, β-secretase, widely known as β-site APP cleaving enzyme 1 (BACE1), has also been identified as a prime therapeutic target for AD intervention. Its inhibition would halt the formation of Aβ at the first step of APP amyloidogenic processing. The therapeutic potential of BACE1 has been confirmed. In this regard, it has been reported that genetic inhibition of the enzyme rescues memory deficits in AD model animals^17^, and BACE1-deficient neurons fail to secrete Aβ peptides or generate β-C terminal fragment (β-CTF)^18^. In view of these strong *in vivo* and *in vitro* validations of critical roles for BACE1 in Aβ generation and AD pathology, intense efforts are underway in academia and industry to develop potent inhibitors of BACE1. Most of the early BACE1 inhibitors were non-cleavable peptide-based transition state analogues modeled after the β-secretase cleavage site of APP^19^. Unfortunately, while these peptidomimetic BACE1 inhibitors show dramatic impacts on Aβ generation *in vitro*, the majority of these inhibitors tend to possess poor drug-like properties *in vivo*, due to poor oral bioavailability, short serum half-life or low blood-brain barrier (BBB) penetration. More recently, a number of non-peptidomimetic candidates for BACE1 inhibitors have been discovered, including carbinamines, acylguanidines, aminoquinazolines and aminothiazines^20^,^21^,^22^,^23^. Moreover, the poor BBB penetration has been solved with the development of potent third-generation small-molecule BACE1 inhibitors that exhibit satisfactory pharmacokinetic profiles and robust cerebral Aβ reduction in preclinical tests^24^,^25^. Several of these BACE1 inhibitors have entered clinical trials, including MK8931 (Merck), LY2886721 (Eli Lilly and Co.) and E2609 (Eisai)^26^.

Compared to γ-secretase, initial reports have indicated that BACE1-null mice were viable, fertile, and devoid of abnormalities, suggesting that inhibition of this enzyme could be clinically feasible with few mechanistic side effects^27^,^28^. However, subsequent investigations found that BACE1 is also a multi-substrate enzyme and identified more than several abnormalities in BACE1-null mice^26^,^29^. Although these BACE1-null abnormalities are relatively mild, they are complex neurological phenotypes that raise a concern that complete inhibition or entire absence of BACE1 function may not be free of mechanism-based side effects. In fact, a phase II clinical trial of LY2886721 (the promising inhibitor of BACE1 by Eli Lilly and Co.) was suspended in June 2013 due to possible liver toxicity^26^,^30^. Thus, while these classes of non-peptide type BACE1 inhibitors utilize novel interactions with both the catalytic machinery and the specificity pockets of BACE1, combining potency, selectivity and the desired safety profiles remains to be a continued challenge. Hence, there is still a clear need for a novel biochemical research for development of potent and selective BACE1 inhibitors with properties optimal for central nervous system therapeutics.

To address this need, we designed a novel substrate-based peptidomimetic BACE1 inhibitor, termed human Swedish mutant APP (APPswe) BACE1 binding site peptide (APPsweBBP). This peptide is a 12-AA-residue fragment derived from human APPswe, containing APPswe β-cleavage sites (Glu665–Arg676 of APPswe770 isoform). Since APPswe has higher affinity for BACE1 compared to human wild-type APP (APPwt), we hypothesized that BACE1 would preferentially proteolyze APPswe as opposed to APPwt. To exclude the epitope of Aβ being produced, we constructed APPsweBBP to lack the intact Aβ domain. In order to develop this specific modulator against BACE1-mediated APP cleavage, we screened peptides derived from the APPswe or APPwt genes, and found that APPsweBBP is the most effective BACE1 inhibitor using a cell-free assay. In order to improve BBB permeability of APPsweBBP, the peptide was further conjugated with the membrane fusion fragment of HIV-1 Tat protein, yielding TAT-APPsweBBP^31^,^32^, which has been demonstrated to promote delivery through the cell membrane as well as the BBB^33^,^34^,^35^. The BACE1 inhibitory and anti-amyloidogenic activities of TAT-APPsweBBP were further evaluated both *in vitro* and *in vivo*. These efforts resulted in the discovery of a competitive and selective modulator for BACE1-mediated APP-cleavage that significantly decreases APP-specific BACE1 proteolytic function. Therefore, we hypothesize that TAT-APPsweBBP blocks processing of the endogenous APP by preferentially binding to BACE1, thereby competitively inhibiting APP β-cleavage. This would present the possibility of a novel, safe and specific substrate-based BACE1 modulator for preventing and treating AD.

## Results

### TAT-APPsweBBP binds to BACE1 and competitively inhibits BACE1 activity

**Figure 1:**
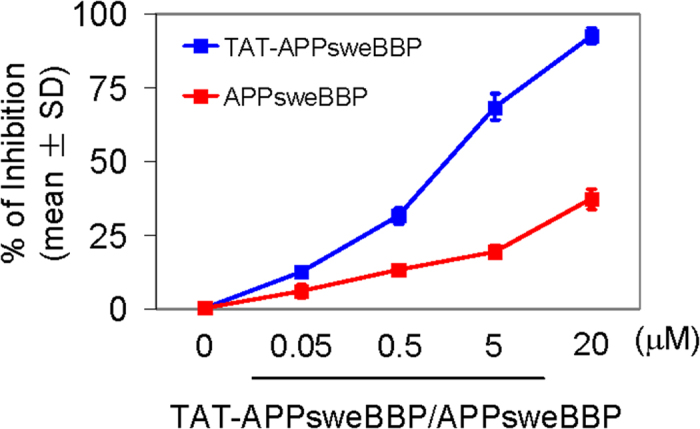
TAT-APPsweBBP competitively inhibits BACE1 activity. BACE1 activity was assayed in the absence and presence of APPsweBBP or TAT-APPsweBBP at 0.05, 0.5, 5 and 20 μM and percentage inhibition of the enzyme was determined.

As a required enzyme for the generation of neurotoxic Aβ from APP, BACE1 is well established as an important mediator of β-amyloid pathology in AD. As such, it has become an important target for disease modifying therapeutics. However, since BACE1 also has important physiological roles, abolishment of the enzyme or its activity may lead to deleterious side effects, as evidenced by failed clinical trials discussed previously. Thus, we endeavored to find an alternative substrate-based inhibitor for BACE1 that does not lead to aberrant amyloidosis. Since previous studies have established a close relationship between α-helix structure and peptide-protein (or protein-protein) interactions^36^,^37^, we had several APP based peptides synthesized that might serve as a preferable substrate for BACE1. Importantly, these peptides were free of the Aβ-containing segment and, thus, cannot be amyloidogenic. In theory, these could be used to “hijack” the BACE1 processing of endogenous APP and lead to a decrease in cerebral Aβ levels. These BACE1 substrates and their controls were incubated with recombinant BACE1, and percent of inhibition of the enzyme was calculated. As shown in Table 1, compared to the APPwt BACE1 binding site peptide (APPwtBBP), the BACE1 inhibiting activity of APPsweBBP was much greater, although the percentage inhibition was still low (~40%). These data suggest that APPswe fragments containing Swedish APP mutant sites exert much higher affinity to BACE1 than their APPwt homologues. Moreover, once the cell penetrating TAT domain was fused with APPsweBBP, yielding TAT-APPsweBBP, its BACE1 inhibitory activity was dramatically elevated (from 38.43 to over−90% inhibition, Fig. 1), and was much higher than the elevation observed with APPwtBBP (elevated by TAT fusion from 16.23 to 21.01%, Table 1). These findings indicate that the α-helical TAT fusion domain may help to promote selective APPsweBBP proteolysis by BACE1, yielding substrate competition against human APPwt.

**Figure 2:**
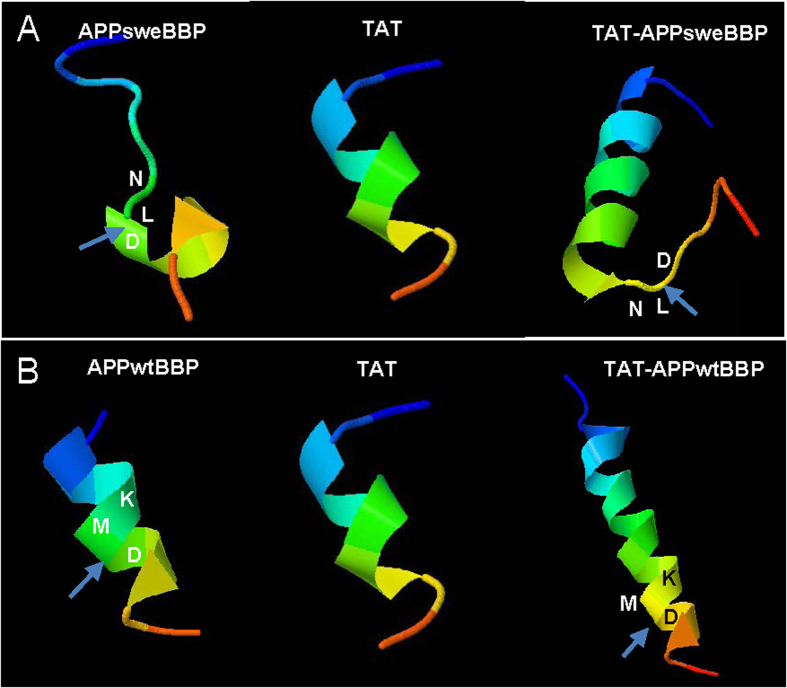
Three-dimensional (3-D) structure analysis predicts that TAT-APPsweBBP strongly and effectively interacts with BACE1. To further predict the potential interactions between TAT-APPsweBBP and BACE1, 3-D structure modeling and enzyme-substrate interactions were analyzed *via* online server Mobyle@RPBS v1.5.1 (http://mobyle.rpbs.univ-paris-diderot.fr/). (**A**) The modeling suggested that after fusion with HIV-1 TAT protein transduction domain, the resulting TAT-APPsweBBP possesses more α-helices than APPsweBBP alone. Moreover, the less folded BACE1 cleavage site of TAT-APPsweBBP outside the α-helix (*L*-*D*, arrow), may create a less sterically hindered and larger site for BACE1 binding, higher binding affinity and more efficient enzymatic digestion compared to TAT-APPwtBBP (*M*-*D*, arrow). (**B**) Owing to more α-helical content, the β-cleavage site of TAT-APPwtBBP is less accessible within the α-helix structure.

These results were further confirmed using three-dimensional (3D) structure modeling *via* the online server Mobyle@RPBS v1.5.1. The results of this analysis (Fig. 2) suggested that after fusion with the HIV-1 TAT protein transduction domain, TAT-APPsweBBP assumes more α-helical structure than APPsweBBP itself, suggesting more potential interaction with BACE1. Furthermore, TAT-APPsweBBP left the BACE1 cleavage site (*L-D*) exposed outside the α helix (Fig. 2A), allowing less sterical hinderance for BACE1 binding and more efficient enzymatic digestion compared to APPsweBBP. In contrast, owing to more α-helical structure, the β-cleavage site (*M*-*D*) of TAT-APPwtBBP is less accessible compared to TAT-APPsweBBP (Fig. 2B). These preliminary findings encouraged us to further evaluate the *in vitro* and *in vivo* efficacy of the newly identified superior BACE1 substrate, TAT-APPsweBBP, which could potentially prevent cerebral amyloidosis with minimal adverse events.

**Figure 3:**
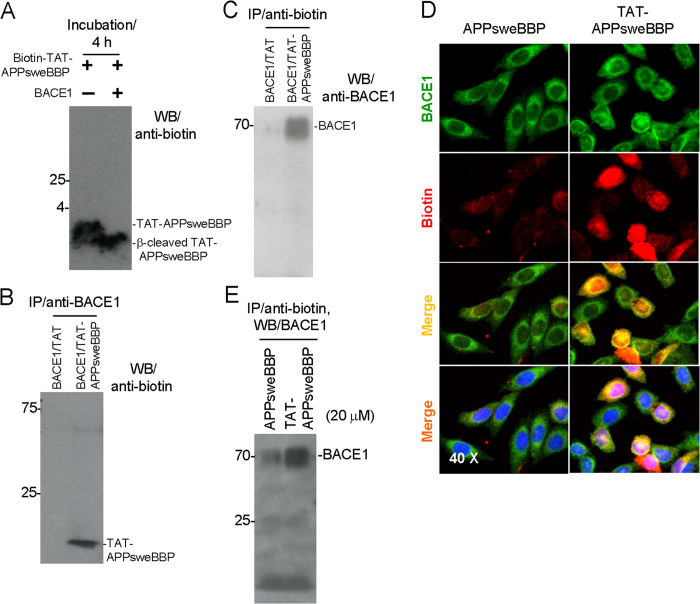
TAT-APPsweBBP strongly binds to and is cleaved by BACE1. (**A**) Biotin-labeled TAT-APPsweBBP was incubated with or without recombinant BACE1 protein and examined by WB. Full length TAT-APPsweBBP and β-cleaved TAT-APPsweBBP were clearly detected. (**B**) Immunoprecipitation (IP) with anti-BACE1 antibody and subsequent WB with anti-biotin antibody and (**C**) IP with anti-biotin antibody and then WB with anti-BACE1 antibody both reveal that TAT-APPsweBBP, but not TAT peptide, binds to BACE1. (**D**) To further confirm TAT-APPsweBPP can bind to BACE1, human wild-type APP expressing Chinese hamster ovary (CHO/APPwt) cells were incubated with biotin-labeled TAT-APPsweBBP or biotin-labeled APPsweBBP at 20 μM for 30 min, and then stained with anti-BACE1 and anti-biotin antibodies. TAT-APPsweBPP showed a strong association with BACE1 in both cytoplasmic membrane and intracellular compartments. (**E**) In addition, these treated cells were further analyzed by IP with anti-biotin and subsequent WB with anti-BACE1 antibodies, with results showing that TAT-APPsweBBP strongly and specifically binds to BACE1.

In order to further confirm the interactions of TAT-APPsweBBP with BACE1, we co-incubated biotin-labeled TAT-APPsweBBP (TAT-APPsweBBP-biotin) with recombinant BACE1 protein for 4 h. Subsequently, Western blot (WB) analysis clearly revealed that BACE1 mediates conversion of full length TAT-APPsweBBP-biotin to the slightly lower molecular weight β-cleaved TAT-APPsweBBP-biotin fragment (Fig. 3A). Furthermore, when immunoprecipitation (IP) was performed using anti-BACE1 antibody, a biotin-labeled TAT-APPsweBBP band was clearly detected on the immunoblotting (Fig. 3B). The reverse order of IP/WB analysis showed similar TAT-APPsweBBP·BACE1 complex formation (Fig. 3C). The possibility that the TAT-APPsweBBP·BACE1 formation was due to the binding of TAT with BACE1 was excluded because the co-immunoprecipitation of biotin-TAT with BACE1 was not found. IP analysis of the mixture with anti-BACE1 IP, followed by anti-biotin WB (Fig. 3B) or anti-biotin IP followed by anti-BACE1 WB (Fig. 3C) revealed that TAT-APPsweBBP binds to BACE1 protein, while such binding was not observed with biotin-labeled TAT peptide alone. Together, TAT-APPsweBBP associates with and is cleaved by BACE1.

### TAT-APPsweBBP inhibits Aβ generation *in vitro*

Next, we tested the ability of TAT-APPsweBBP to interact with BACE1 *in vitro*. Chinese hamster ovary (CHO) cells engineered to express human APPwt (CHO/APPwt cells) were incubated with biotin-labeled TAT-APPsweBBP or APPsweBBP at 20 μM for 30 min, followed by staining with anti-BACE1 and anti-biotin antibodies. We found that TAT-APPsweBBP much more strongly co-localized with BACE1 protein compared to APPsweBBP in both cytoplasmic membrane and intracellular compartments (Fig. 3D). Subsequently, IP analysis of the cell lysates corroborated these findings, as TAT-APPsweBBP was markedly bound to BACE1 compared to APPsweBBP (Fig. 3E).

**Figure 4:**
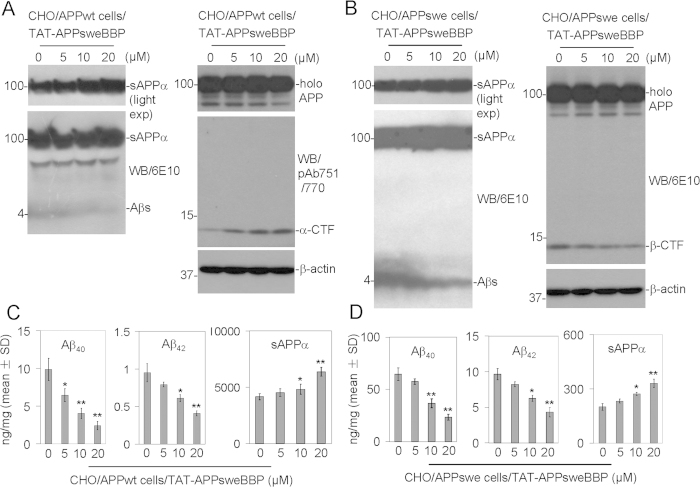
TAT-APPsweBBP inhibits APP amyloidogenic processing. Chinese hamster ovary cells expressing wild-type human APP (CHO/APPwt) or Swedish mutant human APP (CHO/APPswe) were treated with TAT-APPsweBBP for 24 h. Cultured media and cell lysates were then prepared for APP processing analysis by WB and ELISA. (A-B) TAT-APPsweBBP markedly inhibited Aβ_1−40/42_ (A and B, *left panels*) and β-CTF (B, *right panel*), while promoting α-CTF (A, *right panel*) and sAPPα production (A and B, *left panels*), in a dose-dependent manner in both CHO/APPwt (**A**) and CHO/APPswe cells (**B**), while leaving holo APP expression unaltered (A and B, *right panels*), as determined by WB analysis. Light exposure (light exp) more clearly reveals sAPPα band as examined by 6E10 WB analysis. (**C-D**) Consistent with these results, TAT-APPsweBBP significantly decreased Aβ_1−40/42_ and increased sAPPα levels in a dose-dependent manner in both CHO/APPwt (**C**) and CHO/APPswe cells (**D**), as determined by ELISA (*P < 0.05, **P < 0.01). These results are representative of three independent experiments with each condition triplicated.

Since we had identified a potent BACE1 substrate and established that it co-localized with the enzyme *in vitro*, we proceeded to test the therapeutic efficacy of TAT-APPsweBBP for reducing amyloidogenic processing of APP. CHO/APPwt and CHO cells engineered to express human APPswe (CHO/APPswe cells) were cultured separately and treated with TAT-APPsweBBP for 24 h, followed by WB analysis of APP processing in cell lysates and media. Indeed, TAT-APPsweBBP potently opposed amyloidogenic processing of APP by BACE1 in a dose-dependent fashion in both CHO/APPwt and CHO/APPswe cells, evidenced by markedly decreased Aβ_s_ (Fig. 4A,B, *left panels*) and β-CTF production (Fig. 4B, *right panel*), together with increased α-CTF (Fig. 4A, *right panel*) and sAPPα production (Fig. 4A,B, *left panels*). These effects were further supported by Aβ_1−40/42_ and sAPPα enzyme-linked immunosorbent assay (ELISA) of CHO/APPwt (Fig. 4C) and CHO/APPswe cells (Fig. 4D). As expected, however, the CHO/APPswe cells produced comparatively greater levels of Aβ_1−40/42_ and lesser levels of sAPPα than CHO/APPwt cells, as the Swedish mutation possesses greater affinity for BACE1.

### TAT-APPsweBBP crosses the BBB, penetrates into cells and improves AD-like behavioral impairments and pathological changes

Considering that we had identified a potent BACE1 substrate and established its anti-amyloidogenic efficacy *in vitro*, we next evaluated its efficacy *in vivo*. For these experiments, we utilized a 5XFAD transgenic mouse model of AD, which have five familial AD mutations (three human APP and two human PS1 mutations) as first described by Oakley *et al.*^38^. These mice exhibit typical hallmark of AD pathology by 2 months of age, including Aβ aggregates, neurodegeneration, neuronal loss and significant behavioral deficits by 4 to 5 months of age, at an accelerated rate, which makes them ideal for rapidly assessing the efficacy of potential AD therapeutics.

**Figure 5:**
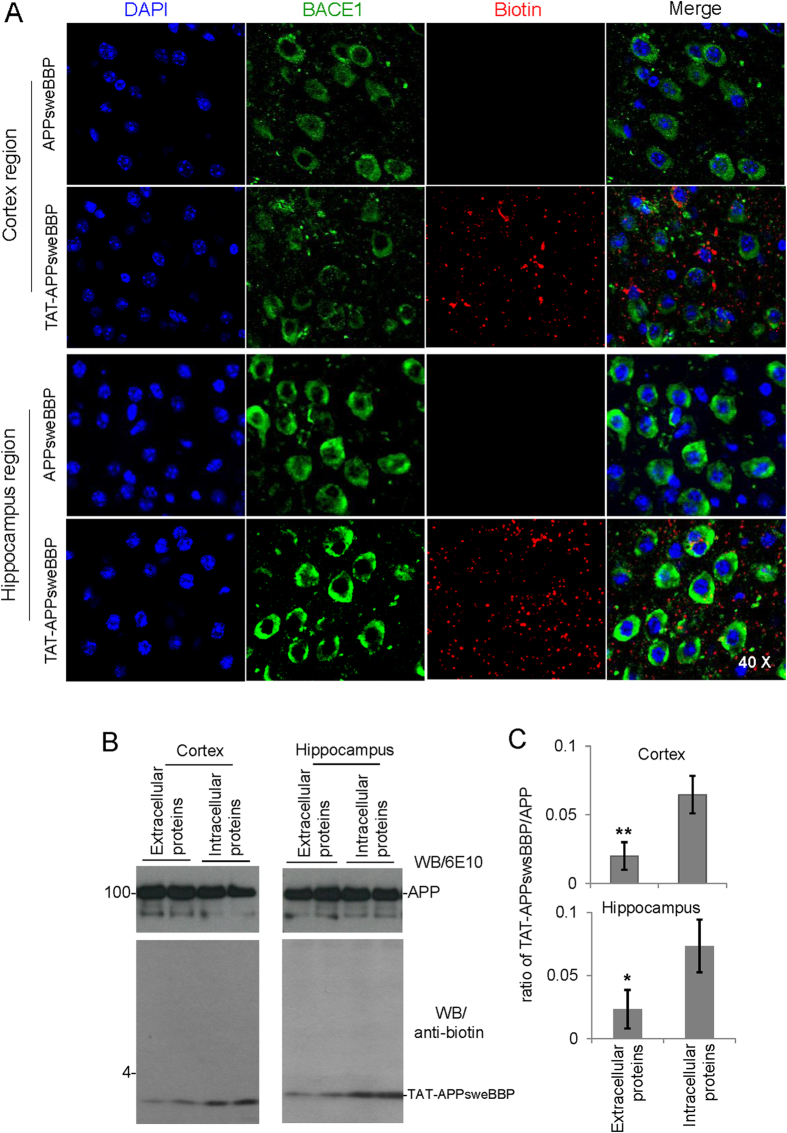
TAT-APPsweBBP crosses the blood brain barrier (BBB) and penetrates into cytoplasm after intraperitoneal (i.p) administration in 5XFAD mice. In order to determine the BBB permeability of TAT-APPsweBBP, biotin-labeled TAT-APPsweBBP or APPsweBBP (100 nmol/kg in 100 μL PBS) was intraperitoneally (i.p.) administered daily to 5XFAD mice at 2 months of age for 5 days (*n* = 5, female). The mice were euthanized 4 h after the last injection and then brain tissues were removed, sectioned and stained with anti-BACE1 and anti-biotin antibodies. Alexa Fluor® 594 donkey anti-rabbit IgG was used to detect the biotin signal and Alexa Fluor® 488 goat anti-mouse IgG was used to detect the BACE1 signal. All images were taken with an Olympus Fluoview FV1000 laser scanning confocal microscope (**A**). Results show that TAT-APPsweBBP is more permeable across the BBB and more highly associates with BACE1 compared to APPsweBBP. No noticeable differences in biotin distribution were observed between 5XFAD and WT control mice following either biotin-labeled TAT-APPsweBBP or APPsweBBP injection (data not shown). In addition, for further confirming the preferred penetrating of TAT-APPsweBBP across cellular membrane into cytoplasm, either extracellular of intracellular TAT-APPsweBBP levels were detected by WB. The data clearly demonstrated that TAT-conjugation facilitate the peptides to enter into cytoplasm of brain cells as indicated by a higher intracellular TAT-APPsweBBP distribution (**B** and **C**). *P < 0.05, **P < 0.01, compared with intracellular proteins.

We initially tested the hypothesis that TAT-APPsweBBP would more effectively cross the BBB compared to APPsweBBP alone. 5XFAD mice at 2 months of age (*n* = 5, female) were treated intraperitoneally with biotin-labeled TAT-APPsweBBP or APPsweBBP (100 nmol/kg) daily for 5 days and then euthanized 4 h after the last injection. Brain tissues were removed, sectioned and stained with anti-BACE1 and anti-biotin antibodies. Confocal images revealed that the peripherally administered TAT-APPsweBBP indeed highly penetrated the BBB and was detectable in both the cortical and hippocampal regions of the 5XFAD mouse brains, in contrast to peripherally administered APPsweBBP (Fig. 5A). This highlights the importance of TAT-APPsweBBP for cerebral delivery.

Additionally, in order to confirm the TAT-conjugation would facilitate APPsweBBP to penetrate into cells, thereafter to inhibit BACE1 and modulate APP processing, both extracellular and intracellular TAT-APPsweBBP levels in cortex and hippocampus were then detected. As expected, the WB data clearly revealed that TAT-APPsweBBP prefers to enter into cytoplasm of brain cells as indicated by a higher intracellular peptides distribution (Fig. 5B,C).

**Figure 6:**
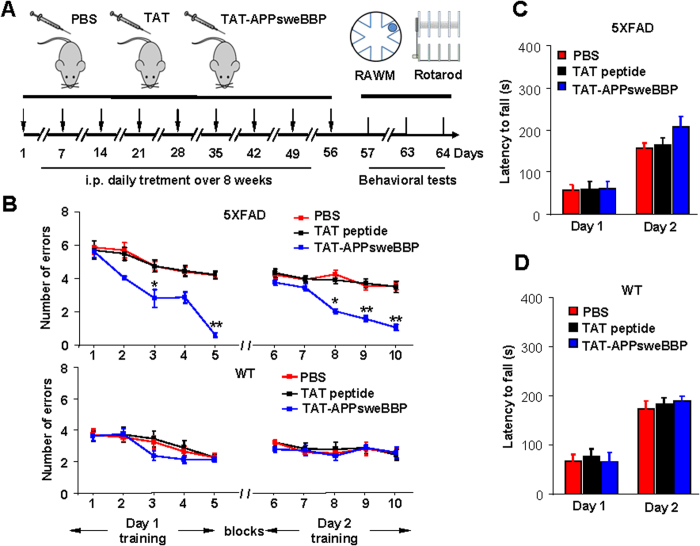
Peripherally administered TAT-APPsweBBP improves hippocampal-dependent learning and memory in 5XFAD mice. In order to correlate the BACE1 inhibitory activities of TAT-APPsweBBP with improved cognitive function, 5XFAD and WT control mice were randomized into three treatment groups: (1) TAT-APPsweBBP treated, (2) TAT peptide-treated and (3) PBS-injected mice (*n* = 10 per treatment, 5 female/5 male). The mice were treated with TAT-APPsweBBP, TAT-peptide (100 nmol/kg in 100 μL PBS) or PBS (100 μL) i.p. daily for 8 weeks. Following the treatment, hippocampal-dependent behavioral learning and memory was assessed with the radial arm water maze (RAWM, schedule as illustrated in (**A**). Cognitive ability was assessed as the number of entry-arm errors before finding the platform. (**B**) Compared to PBS or TAT-treatments, TAT-APPsweBBP enhanced cognitive ability, as evidenced by fewer errors. In contrast, there was no significant difference between WT treatment groups. (**C, D**) Sensorimotor ability was also assessed using the rotarod test in 5XFAD (**C**) and WT control (**D**) mice after treatment. TAT-APPsweBBP treatment tended to enhance motor activity in 5XFAD mice, as shown by increased latency to fall, but this did not reach a statistical level of significance when compared to either TAT peptide- or PBS-treated mice. All data are presented as mean ± SD (*P < 0.05, **P < 0.01, repeated measures 2-way ANOVA with LSD (**B**) or Bonferroni *post hoc* test (**C** and **D**).

We then endeavored to test the efficacy of TAT-APPsweBBP for opposing behavioral impairments and amyloidogenic pathology in 5XFAD mice. To this end, 5XFAD and WT mice were randomized into three treatment groups as follows: (1) TAT-APPsweBBP-treated, (2) TAT-peptide-treated and (3) PBS-treated (control) mice. Each mouse was treated with TAT-APPsweBBP, TAT or PBS by daily intraperitoneal injection (i.p.) for eight consecutive weeks, and then subjected to the radial arm water maze (RAWM) and rotarod tests (Fig. 6A). In the RAWM test, TAT-APPsweBBP-treated 5XFAD mice committed significantly fewer errors, compared to TAT- and PBS-treated mice (Fig. 6B, *upper panel*), indicating that TAT-APPsweBBP treatment could improve hippocampal-dependent learning/memory. In contrast, WT mice exhibited no difference among the various treatment groups (Fig. 6B, *lower panel*). Although all mice demonstrated increased latency to fall after 2 days of rotarod testing, there were no differences between treatment groups on each day for either 5XFAD or WT mice (Fig. 6C,D). Together, TAT-APPsweBBP treatment did not improve motor performance.

**Figure 7:**
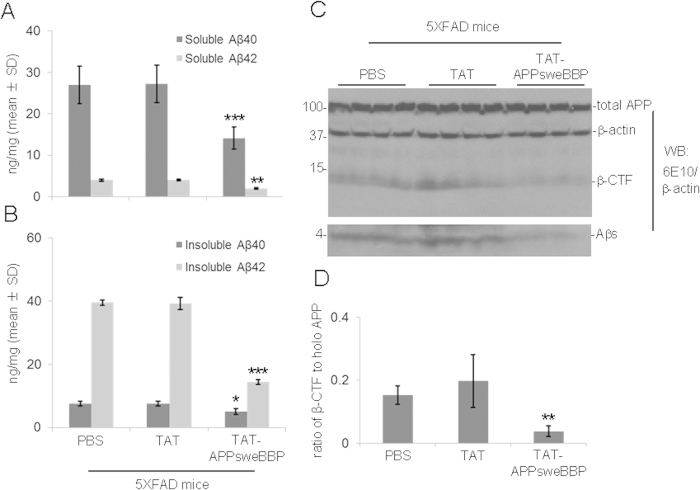
TAT-APPsweBBP markedly inhibits APP amyloidogenic processing in 5XFAD mice. 5XFAD mice were euthanized after 8 weeks of treatment, followed by preparation of brain homogenates for biochemical analyses. TAT-APPsweBBP treatment significantly reduced detergent-soluble (**A**) and insoluble Aβ_1−40/42_ levels (**B**), compared to TAT- or PBS-treatments, as assessed by ELISA (*P < 0.05, **P < 0.01, ***P < 0.005, Student’s *t* test). Data are represented as mean ± SD of Aβ_1−40/42_ (ng/mg of total protein). (**C**) In addition, TAT-APPsweBBP reduced total cerebral soluble Aβ (Aβs) and β-CTF, as determined by WB analysis with 6E10, without significantly altering total APP expression. The blot was re-probed with anti-β-actin antibody. (**D**) Densitometry analysis reveals that TAT-APPsweBBP significantly reduces the band density ratios of β-CTF to holo-APP, compared to TAT- and PBS-treatments (**P < 0.01). Data are represented as mean ± SD.

In addition to behavioral testing, we also evaluated neuronal APP proteolysis by ELISA and WB analysis. Given our findings *in vitro* that TAT-APPsweBBP exerted BACE1 inhibitory and anti-amyloidogenic effects, we hypothesized that we would see similar results in 5XFAD mice. ELISA analysis revealed statistically significant decreases in both detergent-soluble and -insoluble Aβ_1−40/42_ levels in TAT-APPsweBBP-treated 5XFAD mice compared to TAT peptide- or PBS-treated 5XFAD mice (Fig. 7A,B). Moreover, WB analysis indicated that TAT-APPsweBBP-treated 5XFAD mice showed much lower levels of APP amyloidogenic processing products, such as β-CTF and Aβs, compared to TAT peptide- and PBS-treated mice without notable alteration on total APP expression (Fig. 7C,D). In addition, we examined the BACE1, ADAM10 and ADAM17 expressions in brain homogenates of these mice by WB analysis, and found comparable expression levels of these three secretases among TAT-APPsweBBP-, TAT- and PBS-treated 5XFAD mice (Fig. S1).

**Figure 8:**
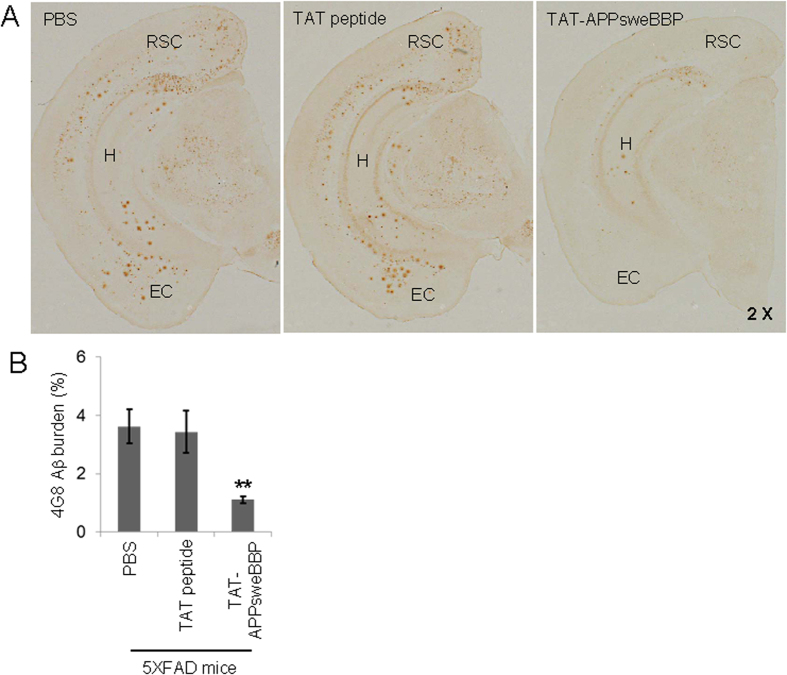
TAT-APPsweBBP markedly reduces β-amyloid deposits. (**A**) TAT-APPsweBBP markedly reduced β-amyloid plaques in retrosplenial cortex (RSC), entorhinal cortex (**EC**) and hippocampus (H) regions of 5XFAD mouse brains, as determined by immunohistochemical staining with anti-Aβ_16−26_ antibody (4G8). (**B**) Percentage of 4G8 immunoreactive areas in regions of interest (RSC, EC and H regions) was quantified by image analysis for each treatment group. Data are represented as mean ± SD (*n* = 10, 5 female/5 male). A *t* test for independent samples revealed significant differences between TAT-APPsweBBP and TAT peptide treatment groups (**P < 0.01), but no significant difference between TAT peptide and PBS groups.

Since cerebral β-amyloid deposits have been a common neuropathological finding in brains with AD, we also studied the effects of peripheral TAT-APPsweBBP treatment on this AD-like pathology phenotype in 5XFAD mice. Immunohistochemical staining with anti-Aβ_16_ antibody (4G8) indicated a marked reduction of β-amyloid plaques in retrosplenial cortex (RSC), entorhinal cortex (EC) and hippocampus (H) regions of 5XFAD mouse brains (Fig. 8A). Moreover, image analysis revealed that the percentage of 4G8 Aβ antibody immunoreactive areas in regions of interest (RSC, EC and H regions) were greatly reduced in the TAT-APPsweBBP-treated 5XFAD mice compared to the TAT- or PBS-treated groups (Fig. 8B).

## Discussion

BACE1 has been found in a variety of tissues throughout the body, while the majority of its expression is shown in the brain^39^. The importance of BACE1 and its influence on AD pathogenesis has been investigated thoroughly since it was first identified in 1999^40^,^41^. APP cleavage by BACE1 produces soluble Aβ fragments, which has the ability to aggregate and migrate onto the dendrites and cell bodies of neuronal cells, initiating chronic immune responses of inflammation and microglial activation. Without early identification and effective inhibition of this pathogenic pathway, the disease is anticipated to become more widespread with the current rapid increase in prevalence within the elderly. Our recent study has suggested an endogenous negative-feedback mechanism whereby the proteolytic product of non-amyloidogenic processing of APP, sAPPα, inhibits BACE1-mediated amyloidogenic APP cleavage in a mouse model^42^. This scenario resulted in an overall reduction in Aβ formation under physiological conditions. Importantly, the consequence of the BACE1 proteolytic pathway being impaired in any way could result in a decrease in APP amyloidogenic processing.

Attempts to inhibit BACE1 have been relatively fruitless with therapeutic trials being aborted in the early stages^26^. A number of obstacles (*i.e.*, those relating to solubility, bioavailability, potency and effectiveness) must be overcome in the development of such inhibitors. Of note, there are also a number of substrates cleaved by BACE1 in addition to APP, regulating voltage gated sodium channels and axon myelination as examples^43^,^44^. In this regard, it has reported that BACE1-null mice exhibit hypomyelination, mediated by reduced neuregulin-Akt signaling and myelinating protein levels^44^. This can create adverse reactions beyond the targeted anti-amyloidogenic effects by BACE1 inhibitors^26^,^29^. Thus, newly generated inhibitors of BACE1 with novel therapeutic strategies were required.

Our current findings suggest that manipulation of BACE1 with natural peptides as opposed to synthetic chemical inhibitors may be a novel and safe strategy for BACE1 targeted therapeutics (Fig. S2). Furthermore, with the roles of BACE1 in proteolysis of many natural substrates, complete inhibition of BACE1 would result in undesirable off-target effects. This would be especially true in light of the fact that APP may not even be the primary substrate of this enzyme^25^. Therefore, specific inhibition of only APP β-cleavage, but not complete inhibition of BACE1 function, may be the most appropriate therapy to normalize the increased APP-directed BACE1 activity seen in AD patients.

In our present study, a cell-free BACE1 activity assay clearly indicated APPsweBBP competitively inhibits the cleavage of commercial BACE1 substrate (EVNLDAEFK) by BACE1 (Table 1). Moreover, TAT-APPsweBBP showed much higher inhibiting activity than its prototype peptide (APPsweBBP) (Fig. 1), indicating a positive role of the TAT conjugation in BACE1 inhibition. Data from IP and WB analyses also indicated that, after binding with BACE1 (Fig. 3B,C), TAT-APPsweBBP can also be cleaved (Fig. 3A), indicating that TAT-APPsweBBP elicits a functional inhibition of BACE1 in contrast to recently developed non-cleavable substrate-based peptidomimetics. TAT-APPsweBBP treatment reduced BACE1-mediated APP amyloidogenesis both *in vitro* (Fig. 4) and in 5XFAD mice (Fig. 7), while also reducing cognitive impairments (Fig. 6). In addition, TAT-APPsweBBP did not alter myelin signaling, observed as unchanged Akt phosphorylation and myelin basic protein levels (Fig. S3), confirming that TAT-APPsweBBP specifically reduces BACE1 activity without eliciting side effects.

It should be noted that a previous study reported a series of BACE1 inhibitors designed from substrate-based peptide sequences to competitively inhibit its binding regions and shut off its enzymatic properties^45^. The initial peptide sequences showed promise with high potency and selectivity but did not progress as a viable pharmaceutical target, because they have large molecular weights and are unstable. Indeed, one of these peptides, OM99-2 (EVNLA*AEF), was reported to have a molecular weight (>18 amino acids residues) that was too large to cross the BBB and also to be not stable enough for use in therapeutic trials^19^, even though the peptide did have a high level of potency. These studies suggest that substrate-based anti-BACE1 peptide-therapeutics may fulfill the desired properties required for an inhibitor, with the only issue being BBB transport. This has motivated the current research into small substrate-based β-cleavage specific BACE1 inhibitors that can freely penetrate the BBB.

Indeed, better BBB penetration creates a challenge that goes beyond the production of protein specific intervention strategies. In particular, the drug needs to maintain an ability to inhibit BACE1, maintain solubility and cross the tight junctions of the cerebral vascular endothelium. To overcome this challenge, the HIV-1 TAT fusion domain, which has been demonstrated to favor intracellular delivery and efficient penetration through the BBB^46^, was conjugated to our novel competitive substrate-based BACE1 inhibitor. We hypothesized that conjugation of this α-helical peptide to our BACE1 inhibitor would not only favor its crossing the BBB but also favor it’s binding with BACE1 (Fig. S4). As shown in the previous studies, a close relationship exists between the α-helical structure and peptide-protein (or protein-protein) interactions^36^,^37^. The 3D structure prediction in our present study (generated by Mobyle@RPBS v1.5.1) suggested that TAT-conjugation indeed enhances the α-helical structure of APPsweBBP (Fig. 2), while our cell-free assay indicated that this conjugation enhances the inhibitory potency of this peptide (Fig. 1). *In vivo* study further supported our hypothesis, as evidenced by greater distribution of TAT-APPsweBBP than APPsweBBP into cortical and hippocampal regions after 5-days systemic administration (Fig. 5).

Taken together, the present report suggests that we can use the higher affinity of APPswe for the design of a peptide (APPsweBBP) as the competitive and specific inhibitor at the BACE1 APP cleavage site. By coupling this peptide to TAT, we found that TAT-APPsweBBP exhibits enhanced efficacy for BACE1 inhibition as well as enhanced BBB penetration, decreasing Aβ production both *in vitro* and *in vivo.* Underlying mechanisms may be a conformational change promoted by fusion of APPsweBBP with the TAT fusion domain, thereby favoring interaction of APPsweBBP with BACE1. Since TAT-APPsweBBP lacks the intact Aβ domain, it “hijacks” endogenous APPwt binding to BACE1 due to its greater affinity^47^,^48^, thereby reducing the cleavage of endogenous APPwt without concurrent Aβ generation. Although future studies should confirm that no other substrates of BACE1 are affected by TAT-APPsweBBP, our current findings provide evidence for a novel strategy for alternative design of BACE1 inhibitors and suggest that TAT-APPsweBBP may be a novel, safe and effective APP substrate-based BACE1 inhibitor for the treatment of AD.

## Methods

### Peptide synthesis and antibodies

APPsweBBP is a truncated fragment of APPswe spanning 12 amino acid residues (Glu665-Arg676). TAT, an 11-amino acid-residue protein-transduction domain derived from the HIV-1 transactivator of transcription protein, was conjugated at the C-terminus of APPsweBBP and biotin was conjugated at the N-terminus. All peptides sequences in the present study (Table 1) were commercially synthesized by GenScript Corporation (Piscataway, NJ). Antibodies include: anti-BACE1 monoclonal antibody (Merck Millipore, Darmstadt, Germany), anti-biotin polyclonal antibody (Cell Signaling, Beverly, MA), anti-N-terminal Aβ monoclonal antibody (6E10, Covance, Emeryville, CA), anti-Aβ_16_ monoclonal antibody (4G8, Covance), anti-APP C-terminal polyclonal antibody (pAb751/770, EMD Biosciences, La Jolla, CA), anti-ADAM10 polyclonal antibody (Merck Millipore), anti-ADAM17 monoclonal antibody (TACE; Sigma-Aldrich, St Louis, MO), anti-Akt and phospho-Akt^Ser473^ monoclonal antibodies (Cell Signaling), anti-myelin basic protein (MBP) polyclonal antibody (Sigma-Aldrich) and anti-β-actin monoclonal antibody (Sigma-Aldrich).

### BACE1 activity assay

The BACE1 inhibiting activity of APP-based BACE1 site binding peptides were determined using a fluorescence resonance energy transfer (FRET) assay (Pan Vera Co., Madison, WI), which employs a recombinant baculovirus-expressed BACE1 and a specific substrate (Rh-EVNLDAEFK-quencher) that is based on the APPswe mutation. This specific peptide substrate becomes highly fluorescent upon enzymatic cleavage. A mixture of 10 μL of BACE1 (1.0 U/mL in 50 mM Tris, pH7.5, 10% glycerol), 10 μL of FRET-substrate, and 10 μL of the various APP-based BACE1 site binding peptides (final concentration 20 μM) were incubated for 90 min in the dark at room temperature. Then, 10 μL of BACE1 stop buffer (2.5 M sodium acetate) was added to the mixture. Fluorescence was read using a fluorometer, with excitation at 545 nm and emission at 585 nm.

In order to confirm that APPsweBBP containing the β-cleavage site could bind to and be consequently proteolyzed by BACE1, 10 μg of biotin-labeled TAT-APPsweBBP was incubated with or without 10 μL of recombinant BACE1 protein (1.0 U/mL) in BACE1 reaction buffer for 4 h at 37 °C and then subjected to WB analysis as described previously^31^,^42^. In addition, we performed immunoprecipitation (IP) and WB analyses of this mixture with anti-BACE1 and anti-biotin antibodies to confirm that TAT-APPsweBBP, but not TAT, specifically binds to BACE1.

### Cell culture

Chinese hamster ovary (CHO) cells engineered to express human APPwt (CHO/APPwt cells) or human APPswe (CHO/APPswe cells) were kindly provided by Dr. Stefanie Hahn and Dr. Sascha Weggen (University of Heinrich Heine, Dusseldorf, Germany). As described in our recent report^42^, these cells were cultured in Dulbecco’s modified Eagle’s medium with 10% fetal bovine serum, 1 mM sodium pyruvate and 100 U/mL of penicillin/streptomycin.

To confirm the binding of APPsweBBP with BACE1, CHO/APPwt cells were plated in 8-well chambers (5×10^5^/well), incubated with TAT-APPsweBBP-biotin or APPsweBBP-biotin at 20 μM for 30 min, fixed with 4% paraformaldehyde in 0.05 M PBS for 20 min, washed and permeabilized with 0.5% Triton X-100. Following application of blocking buffer, these cells were stained with mouse anti-BACE1 and anti-biotin antibodies overnight at 4 °C for immunocytochemical analysis. In order to determine the inhibitory effects of TAT-APPsweBBP on BACE1-mediated APP β-cleavage and subsequent Aβ production, both CHO/APPwt and CHO/APPswe cells were plated in 24 well-plates (3×10^5^/well) and treated with TAT-APPsweBBP at 0 to 20 μM for 24 h. The cultured media were collected for Aβ and sAPPα analysis by ELISA and WB. Cell lysates were also prepared for analysis of APP amyloidogenic processing by WB.

### 5XFAD Mice

Transgenic AD mice [5XFAD; B6SJL-Tg(APPSwFlLon,PSEN1*M146L*L286V) 6799Vas/Mmjax] at 2.5 months of age were obtained from the Jackson Laboratory (Bar Harbor, ME) and housed and maintained in the Animal Facility of College of Medicine at University of South Florida (USF). The mice at 3.5 months of age were treated with TAT-APPsweBBP, TAT peptide (100 nmol/kg in 100 μL PBS) or PBS (100 μL) daily by intraperitioneal injection (i.p.) for eight consecutive weeks. The mice were subjected to behavioral testing after 8-week treatment and then euthanized for immunohistochemical, WB and ELISA analyses. Animals were housed and maintained at USF and all experiments were in compliance with protocols approved by USF Institutional Animal Care and Use Committee. All methods were performed in accordance with relevant guidelines and regulations.

### Behavioral Tests

Spatial learning and memory performance was tested for each mouse using the Two-day Radial Arm Water Maze (RAWM) test^49^. The RAWM was carried out in a 100-cm circular pool with six swim alleys radiating from a common circular swim area. An escape platform was placed near the end of one arm, which forces mice to use working memory to perform this task. On day 1, mice are trained for 15 trials (5 blocks, 3 trials per block), with trials alternating between visible and hidden platform. On day 2, mice are trained for 15 trials with the platform hidden (submerged below the water surface). The platform position remains constant for each mouse. For each trial, the mice were placed in the designated start arm facing the common circular swim area and given 1 min to find the platform. An error was charged each time a mouse entered an incorrect arm. If the platform was not located during the 1-min trial, the mouse was guided to the platform and allowed to remain there for 30 s before the next trial begins. Cognitive ability was assessed as the number of entry-arm errors.

The rotarod task was also performed to exclude the possibility that positive effects of any treatment in RAWM test are due to improvements in sensorimotor ability. Mice were positioned on the rod (diameter 3.6 cm) of the equipment (Rotarod 7650 accelerating model; Ugo Basile, Biological Research Apparatus, Varese, Italy), which was initially set at 1.0 rpm. The rod was then allowed to steadily accelerate up to 40.0 rpm over a 3-min session and evaluation was made by monitoring latency to fall.

### Immunohistochemistry

Fixed cell cultures and brain sections were stained with indicated antibodies and then treated with donkey anti-mouse IgG conjugated with Alexa Fluor 488 (1:200) or goat anti-rabbit IgG conjugated with Alexa Fluor 594 (1:200; Invitrogen, Carlsbad, CA). The slides were then washed, mounted with DAPI medium (Vector Laboratories, Burlingame, CA) and visualized with an Olympus FV1000 confocal microscope (Tokyo, Japan).

For paraffin brain tissues, we sectioned five coronal sections per region with a 100-μm interval and a thickness of 5-μm for retrosplenial cortex (RSC), entorhinal cortex (EC), and hippocampus (H), located at bregma −2.92 to −3.64 mm. Immunohistochemical staining was conducted according to the manufacturer’s protocol using a Vectastain ABC Elite kit (Vector Laboratories, Burlingame, CA) coupled with the diaminobenzidine reaction, except that the biotinylated secondary antibody step was omitted. A biotinylated human Aβ17 −24 monoclonal antibody (4G8; 1:200, Covance Research Products, Emeryville, CA) was used as a primary antibody. Images were acquired as digitized tagged-image format files (to retain maximum resolution) using a BX60 microscope with an attached CCD camera system (DP-70, Olympus, Tokyo, Japan), and digital images were routed into a Windows PC for quantitative analyses using SimplePCI software (Hamamatsu Photonics, Hamamatsu, Shizuoka, Japan). We captured images of five 5-μm sections through each anatomic region of interest (RSC, EC, and H) based on anatomical criteria defined by Franklin and Paxinos^50^, and obtained a threshold optical density that discriminated staining from background. Each anatomic region of interest was manually edited to eliminate artifacts. Selection bias was controlled for by analyzing each region of interest in its entirety.

### Protein extraction

For specific extraction of extracellular versus intracellular proteins, hemibrains were harvested and placed in 500 μl of solution containing 50 mM Tris-HCl, pH 7.6, 0.01% NP-40, 150 mM NaCl, 2 mM EDTA, 0.1% SDS, 1 mM phenylmethylsulfonyl fluoride, and protease inhibitor cocktail (Sigma) as previously describe^51^. Soluble, extracellular proteins were collected from mechanically homogenized lysates after centrifugation for 5 min at 3000 rpm. Cytoplasmic proteins were extracted from cell pellets mechanically dissociated with a micropipettor in 500 μl of TNT buffer (50 mM Tris-HCl, pH 7.6, 150 mM NaCl, and 0.1% Triton X-100) after centrifugation for 90 min at 13,000 rpm. Insoluble material was incubated with 20 μl of 70% formic acid, mechanically dissociated with a micropipette, gently agitated for 1 h, and buffered with 380 μl of 1 M Tris-HCl, pH 8.0. Samples were centrifuged for 90 min at 13,000 rpm, and supernatants were collected for analysis.

### WB analysis

WB analysis was performed as previously described^32^,^45^. Briefly, the proteins from the various cell-free suspensions, cell lysates and brain homogenates were electrophoretically separated using 10% bicine/tris gel (8 M urea) for proteins less than 5 kD or 10% tricine/tris gels for larger proteins. Electrophoresed proteins were transferred to polyvinylidene difluoride membranes (Bio-Rad, Richmond, CA), washed and blocked for 2 h at room temperature in Tris-buffered saline containing 5% (w/v) nonfat dry milk (TBS/NFDM). After blocking, membranes were hybridized for 2 h with various primary antibodies, washed and incubated for 1 h with the appropriate HRP-conjugated secondary antibody in TBS/NFDM. Blots were developed using the luminol reagent (Thermo Fisher Scientific, Waltham, MA).

### ELISA

Mouse brains were homogenized in ice-cold lysis buffer for 30 s using a Minilys tissue homogenizer (Bertin Technologies, Montigny-le-Bretonneux, France) set at high speed, allowed to stand for 15 min at 4 °C, and centrifuged at 15000 rpm for 30 min. Soluble Aβ_1−40/42_ species and sAPPα were directly detected in cultured cell media or brain homogenates using the Aβ_1−40/42_ (Invitrogen) and sAPPα ELISA kits (IBL-America, Minneapolis, MN). Detergent-insoluble total Aβ_1−0/42_ species were detected in brain by extracting pellets in 5 M guanidine HCl buffer, followed by a 1:20 dilution in lysis buffer.

### Statistical Analysis

Data are expressed as mean ± SD. Comparison between groups was performed by Student’s *t* test or one-way ANOVA followed by LSD or Bonferroni *post hoc* test. As for the RAWM test, the data were analyzed by two-way ANOVA repeated measurement (treatment × time). Separate pair-wise comparisons were run to determine which trials were different between groups when a significant overall group difference was found. *P* < 0.05 was considered statistically significant.

## Additional Information

**How to cite this article**: Li, S. *et al.* Swedish mutant APP-based BACE1 binding site peptide reduces APP β-cleavage and cerebral Aβ levels in Alzheimer’s mice. *Sci. Rep.* **5**, 11322; doi: 10.1038/srep11322 (2015).

## Supplementary Material

Supplementary Information

## Figures and Tables

**Table 1:**
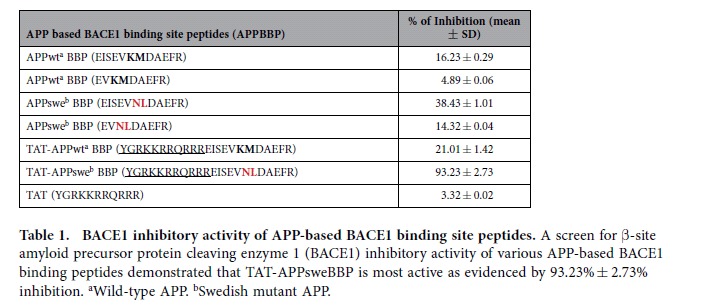
BACE1 inhibitory activity of APP-based BACE1 binding site peptides.
