# Supplementary material for: Swedish mutant APP-based BACE1 binding site peptide reduces APP β-cleavage and cerebral Aβ levels in Alzheimer’s mice
Source: Sci Rep. 2015 Jun 19;5:11322. doi: 10.1038/srep11322 (PMC4473678; doi:10.1038/srep11322)
Supplement: Supplementary Information [file srep11322-s1.pdf]

Supplementary Information

Swedish mutant APP-based BACE1 binding site peptide reduces APP β-cleavage and cerebral Aβ levels in Alzheimer's mice

Song Li[1,2,*], Huayan Hou[1,*], Takashi Mori[3], Darrell Sawmiller[1], Adam Smith[4], Jun Tian[1], Yanjiang Wang[5], Brian Giunta[6], Paul R. Sanberg[4], Sheqing Zhang[1,7] & Jun Tan[1]†

[1]Department of Psychiatry and Behavioral Neurosciences, Morsani College of Medicine, University of South Florida, Tampa, FL 33613, [2]Center for Translational Research of Neurology Diseases, First Affiliated Hospital, Dalian Medical University, Dalian 116011, China, [3]Departments of Biomedical Sciences and Pathology, Saitama Medical Center and Saitama Medical University, Kawagoe, Saitama 350-8550, Japan, [4]Department of Neurosurgery and Brain Repair, Center Of Excellence for Aging & Brain Repair, Morsani College of Medicine, University of South Florida, Tampa, FL 33613, [5]Department of Neurology, Daping Hospital, the Third Military Medical University, Chongqing 400042, China, [6]Neuroimmunology Laboratory, Department of Psychiatry and Behavioral Neurosciences, Morsani College of Medicine, University of South Florida, Tampa, FL 33613, [7]Department of Neurology, Changhai hospital, Shanghai 200433, China.

Fig. S1. BACE1, ADAM10 and ADAM17 expression levels in brain homogenates of TAT-APPsweBBP-, TAT- and PBS-treated 5XFAD mice. The expression levels of BACE1, ADAM10 and ADAM17 were not significantly different between TAT-APPsweBBP-, TAT- or PBS-treated mice, as determined by WB analysis.

Fig. S2. Competitive inhibition of BACE1 endogenous APP cleavage by TAT-APPsweBBP. BACE1 cleaves APP at the β-cleavage site, with higher efficacy for APPswe (EISEVNLDAEFR) than APPwt (EISEVKMDAEFR), yielding β-CTF and sAPPβ. β-CTF is then further cleaved at the γ-site to yield Aβ. TAT-APPsweBBP, a BACE1 binding site peptide derived from APPswe (EISEVNLDAEFR) and fused with TAT transduction domain (YGRKKRRQRRR), competitively blocks BACE1 cleavage of APPwt abrogating Aβ production.

Fig. S3. Total Akt, phosphorylated Akt and MBP expression levels in brain homogenates did not differ between TAT-APPsweBBP-, TAT- and PBS-treated 5XFAD mice. Akt phosphorylation state and MBP expression levels were examined by WB with specific antibodies against total and phosphorylated Akt (**A**) and MBP protein (**B**).

Fig. S4. TAT (47-57) peptide conjugation facilitates APPsweBBP penetration across BBB. Peripherally injected TAT-APPsweBBP freely enters brain microvascular endothelial cells and crosses the BBB from the capillary lumen into the brain parenchyma, while APPsweBBP is stopped at the capillary lumen.

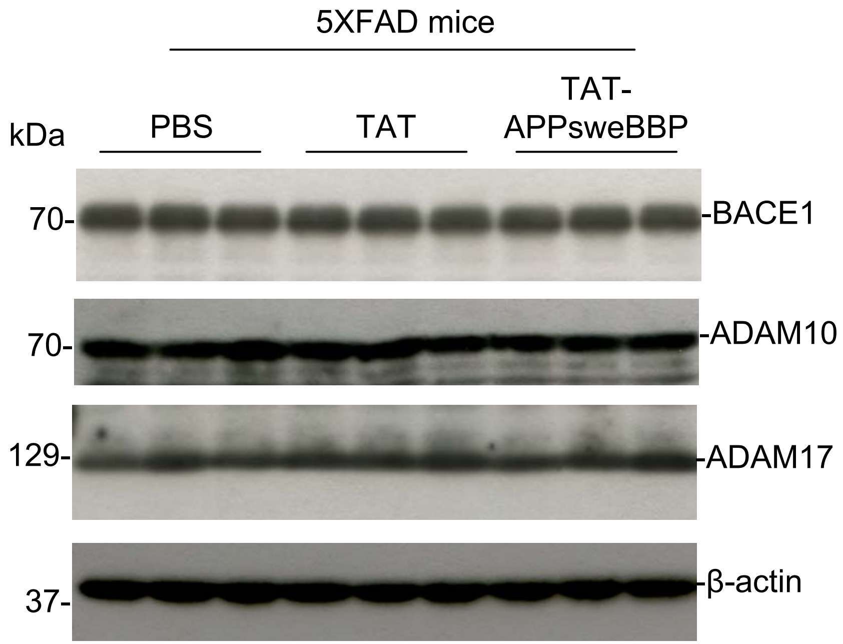

5XFAD mice
kDa
PBS
TAT
TAT-APPsweBBP
70-
-BACE1
70-
-ADAM10
129-
-ADAM17
37-
-β-actin

Fig. S1

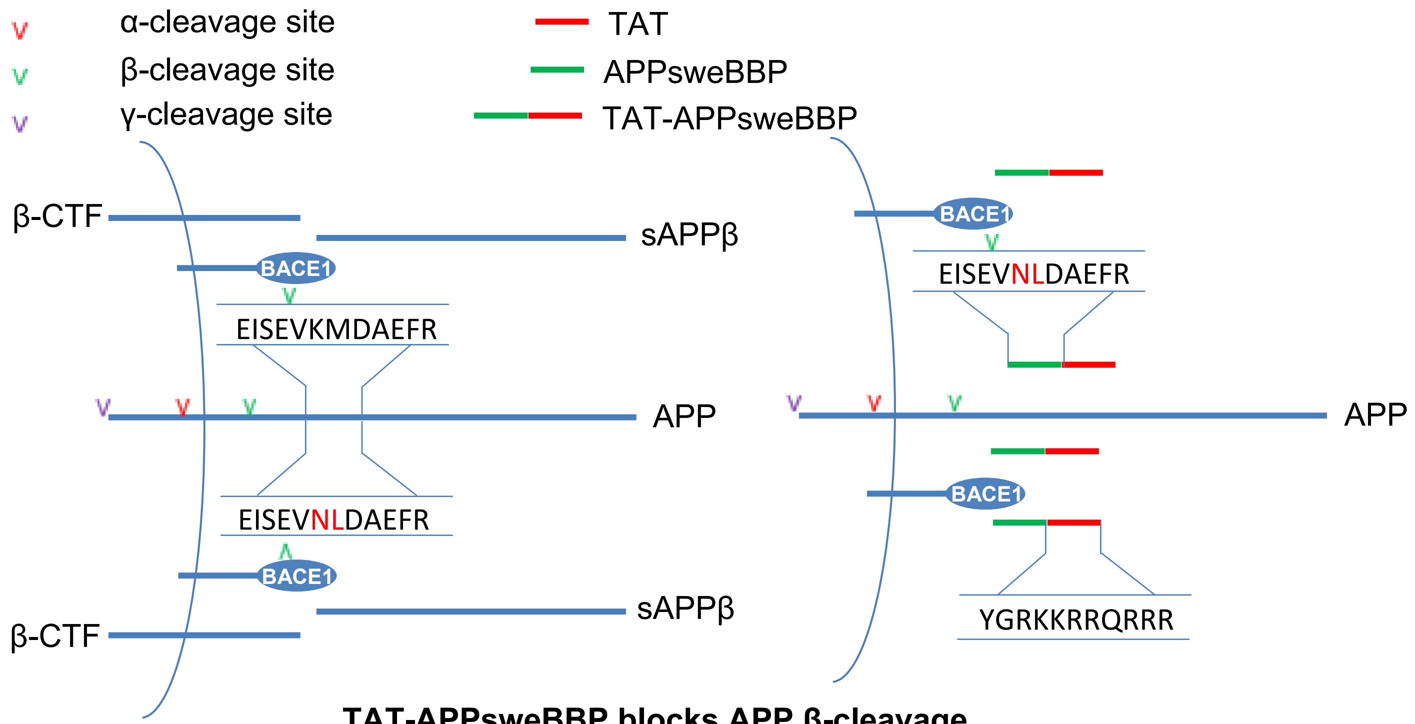

TAT-APPsweBBP blocks APP β-cleavage

Swedish mutant APP β-secretase cleavage binding site peptide**: APPsweBBP (EISEVNLDAEFR)**
TAT transduction domain fused Swedish mutant APP β-secretase cleavage binding site peptide:
TAT-APPsweBBP (YGRKKRRQRRREISEVNLDAEFR)

Fig. S2

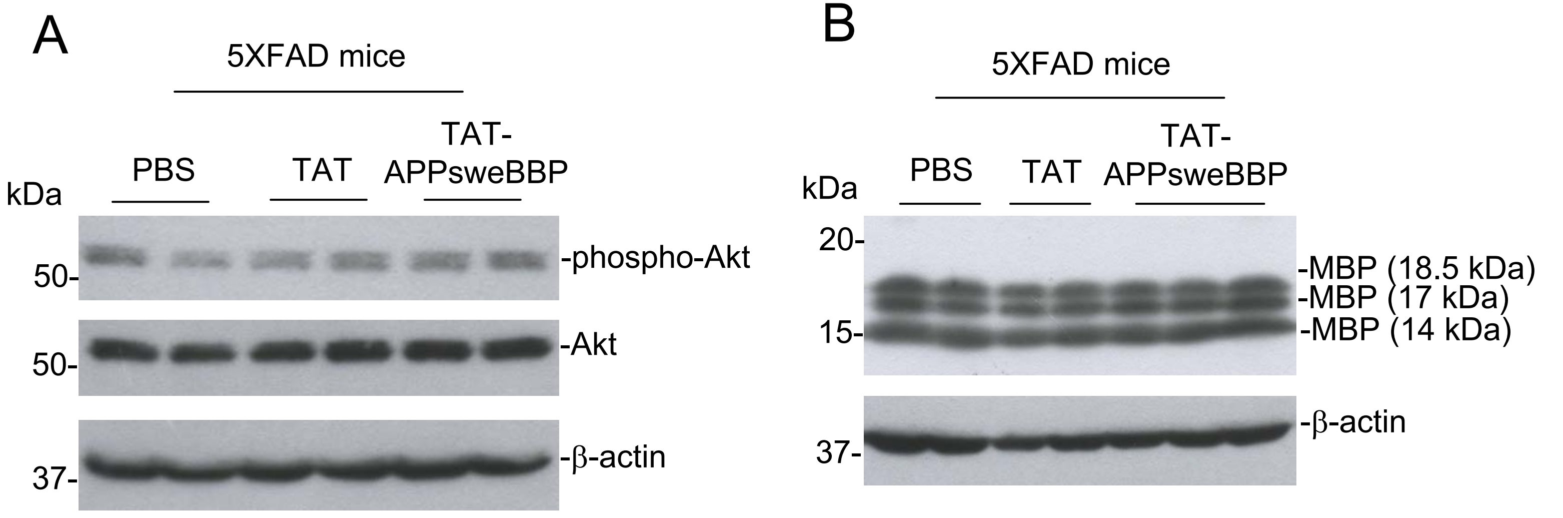
A
5XFAD mice
PBS
TAT
TAT-
APPsweBBP
kDa
50-
50-
37-
-phospho-Akt
-Akt
-β-actin
B
5XFAD mice
PBS
TAT
TAT-
APPsweBBP
kDa
20-
15-
37-
-MBP (18.5 kDa)
-MBP (17 kDa)
-MBP (14 kDa)
-β-actin

Fig. S3

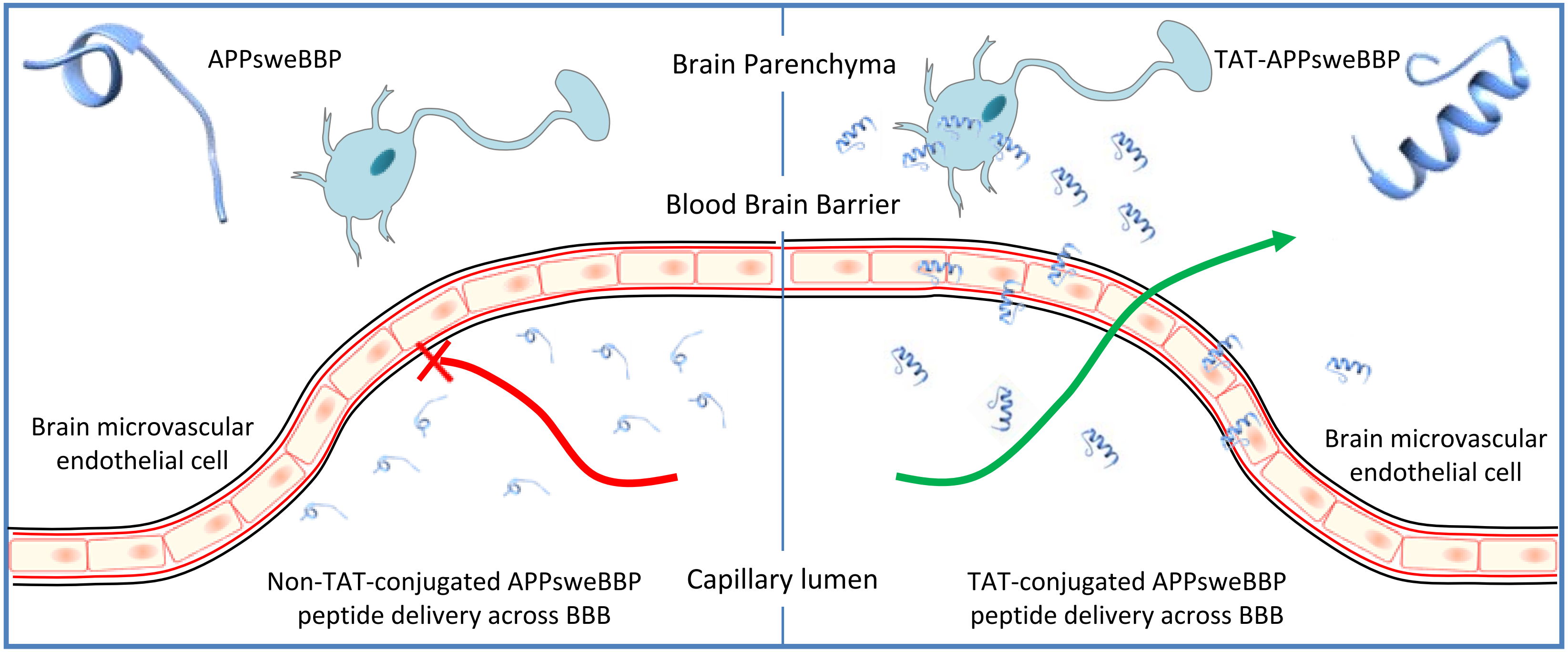
APPsweBBP
Brain Parenchyma
TAT-APPsweBBP
Blood Brain Barrier
Brain microvascular endothelial cell
Brain microvascular endothelial cell
Non-TAT-conjugated APPsweBBP peptide delivery across BBB
Capillary lumen
TAT-conjugated APPsweBBP peptide delivery across BBB

Fig. S4
